# Risk-based diabetes screening in a Hungarian general practice: comparison of laboratory methods and diagnostic criteria

**DOI:** 10.1017/S1463423621000037

**Published:** 2021-04-22

**Authors:** Henrietta Galvács, János Szabó, Zoltán Balogh

**Affiliations:** 1 Department of Nursing, Faculty of Health Sciences, Semmelweis University Hungary, Károly Rácz School of PhD Studies, Semmelweis University, Budapest, Hungary; 2 DRSZF Egészségműhely Ltd, Heves, Hungary; 3 Department of Nursing, Faculty of Health Sciences, Semmelweis University, Budapest, Hungary

**Keywords:** diabetes mellitus, glycated haemoglobin, metabolic syndrome, oral glocuse tolerance test, screening

## Abstract

**Aim:**

Aim of cross-sectional study was to survey the risk of diabetes mellitus in a severely disadvantaged Hungarian community and then to use laboratory tests to screen for potential carbohydrate metabolism disorders among those in the moderate- and high-risk groups.

**Background:**

The prevalence of diabetes mellitus shows a worrisome trend worldwide. Low socio-economic status significantly affects the development of diabetes, healthy life years and life expectancy.

**Method:**

Diabetes risk of the population was surveyed with the FINDRISC (Finnish Diabetes Risk Score) questionnaire, followed by oral glucose tolerance test (OGTT) and glycated haemoglobin test of moderate- and high-risk patients.

**Findings:**

In sample of 551 subjects, moderate or high risk for diabetes was confirmed in 147 patients (26.68%). There was significant correlation between increased risk and age (*P* < 0.001) and between increased risk and body mass index (*P* < 0.001). Significant difference was confirmed between incidences for disease based on the results of OGTT and glycated haemoglobin test when two different criteria systems were used. Age was the strongest predictor of pre-diabetes/diabetes (*P* = 0.016). The presence of metabolic syndrome increased the level of glycated haemoglobin by an average of 0.2% in normal glycemic status.

## Introduction

According to International Diabetes Federation (IDF) estimates, in Hungary, the ratio of adult individuals with diabetes was between 5% and 7% in 2019 (International Diabetes Federation, 2019). Pre-diabetes is a precursor stage of type two diabetes mellitus (T2DM). Pre-diabetes may be present approximately 5 to 15 years before diabetes is diagnosed and certain target organ impairments – primarily macrovascular complications – can develop during this period (Ramlo-Halsted and Edelman, 1999). Moreover, the imbalance in carbohydrate metabolism creates favourable conditions for cancer development (Giovannucci *et al*., 2010; Nakamura *et al*., 2017; Collier *et al*., 2019). Epidemiological research has also confirmed that socio-economic status is a major factor in the development of pre-diabetes and T2DM (Vokó *et al*., 2009; Masseria *et al*., 2010; Kósa *et al*., 2011). Within Europe, Hungary has one of the worst statistics in terms of both mortality and morbidity and poverty is a significant factor. According to the 2017 results published by Eurostat, 25.6% of the Hungarian population lives in poverty (Eurostat, 2018). Our country has some of the highest rates of mortality due to cardiovascular diseases and cancers (lung and colon) within Europe (European Heart Network, 2017; Ferlay *et al*., 2018; Meier *et al*., 2019). The region of Northern Hungary – including the practice chosen by our research group – is one of the most severely disadvantaged area socially, economically and in terms of public health. In Heves County, registered job seekers account for 17% of the economically active population, one of the worst ratios in an already disadvantaged county. One out of every five active participants in the labour market is a registered job seeker and 66% of them have at most a primary school certificate (Municipal Assembly of Heves County, 2018a). The County has a significant Roma population, which is estimated to be approximately 24.4%, and the average age of this population is roughly 10 years younger than that of the non-Roma population (Pénzes *et al*., 2018). In 2016, the raw mortality rate in Heves County was 14.8 per 1000 compared to the national rate of 12.9 per 1000. A review of the mortality statistics of the County shows that in 2016 cardiovascular disease was the most frequent cause of death (768/100 thousand inhabitants), followed by cancer (388/100 thousand inhabitants), and both rates exceeded the national average (Municipal Assembly of Heves County, 2018b). These poor indices are probably due to a lack of health culture and low rates of willingness to participate in screening tests. Based on research data, less than half of the Hungarian population participates in optional screening tests and this rate shows a strong correlation with the socio-economic status of patients (Sándor *et al*., 2018; Hodges *et al*., 2018). These data are a good representation of the socio-economic and public health problems affecting the region. The primary aim of our research project was to analyse the incidence of pre-diabetes as a precursor stage of T2DM in a general practice caring for disadvantaged patients because no relevant statistics for this scenario are available in Hungary. Additionally, since to date no comparative study of diabetes screening methods has been conducted in Hungary, our secondary aim was to determine whether there was a difference in oral glucose tolerance test (OGTT) and glycated haemoglobin (HbA_1c_)-based local and international incidence results. The incidences were calculated separately based on the criteria systems established by the American Diabetes Association (ADA) and the World Health Organization (WHO), respectively. The reason for this was that certain literature does not recommend to use the criteria system established by the ADA among the European population, as it is stricter than that of established by the WHO. Therefore, the WHO and the International Expert Committee (IEC) had previously established an HbA_1c_ range adjusted to their own criteria for the diagnosis of carbohydrate metabolism disorders, which can be used in everyday practice. It is important to note, however, that taking into account the cardiovascular outcomes, HbA_1c_ range recommended by the ADA is proven to be more reliable; also, both cardiology and diabetes associations agree that a fasting glucose level (FPG) above 5.6 mmol/L can be considered as a cardiovascular risk factor (Manley *et al*., 2010; Vistisen *et al*., 2018).

## Methods

### Selection of study subjects

Cross-sectional study was conducted between April 2018 and January 2020 in the general practice of Átány municipality in Heves County. There are approximately 1,500 inhabitants in the municipality. Registered users of the practice, aged 18 years to 75 years, without a history of diagnosed carbohydrate metabolism disorder were included in the study. Pregnant women and people with serious mobility issues were excluded from the study (the latter group was excluded due to the difficulty of travelling to the laboratory site).

### Measurements and qualitative methods

Research was carried out using a two-stage method. In the first stage, diabetes risk was assessed with a FINDRISC (Finnish Diabetes Risk Score) questionnaire, and in the second stage patients with moderate or high risk were referred for OGTT (with 75 grams glucose) and HbA_1c_ tests measured from venous blood. The FINDRISC questionnaires were assessed according to the following classification:

- Less than seven points: low risk,
- 7–11 points: slightly elevated risk,
- 12–14 points: moderate risk,
- 15–20 points: high risk, and
- More than 20 points: very high risk.

Diagnosis of carbohydrate metabolism disorders according to both criteria systems established by the WHO/IEC and the ADA was studied, using both OGTT and HbA_1c_ test. Table 1 shows the two criteria systems (Hungarian Diabetes Association, 2020; American Diabetes Association, 2020).

**Table 1.** Criteria by glycemic status defined by WHO/IEC and ADA

	Laboratory diagnostic criteria by WHO/IEC	Laboratory diagnostic criteria by ADA
**Normal glucose tolerance**		
**FPG**	≤ 6.0 mmol/L	≤ 5.5 mmol/L
**OGTT 2 hours**	< 7.8 mmol/L	
**HbA**_**1c**_	≤5.9%	≤ 5.6%
**Impaired fasting glucose**		
**FPG**	6.1 mmol/L – 6.9 mmol/L	5.6 mmol/L – 6.9 mmol/L
**OGTT 2 hours**	< 7.8 mmol/L	
**HbA**_**1c**_	6.0–6.4%	5.7–6.4%
**Impaired glucose tolerance**		
**FPG**	<7.0 mmol/L	
**OGTT 2 hours**	7.8 mmol/L – 11.0 mmol/L	
**HbA**_**1c**_	6.0–6.4%	5.7–6.4%
**Diabetes mellitus**		
**FPG**	≥7.0 mmol/L	
**OGTT 2 hours**	≥11.1 mmol/L	
**HbA**_**1c**_	≥6.5%	

WHO = World Health Organization; IEC = International Expert Committee; ADA = American Diabetes Association; FPG = fasting plasma glucose; OGTT = oral glucose tolerance test; HbA_1c_ = glycated haemoglobin;

We measured and calculated the anthropometric values of body mass index (BMI), waist circumference and height. BMI was classified according to the WHO criteria presented in Table 2 (World Health Organization, 2006).

**Table 2.** Determination of body mass index based on the World Health Organization criteria

Classification	Body mass index
Underweight	<18.50
Severe thinness	<16.00
Moderate thinness	16.00–16.99
Mild thinness	17.00–18.49
Normal range	18.50–24.99
Overweight	≥25.00
Pre-obese	25.00–29.99
Obese	≥30.00
Obese class I	30.00–34.99
Obese class II	35.00–39.99
Obese class III	≥40.00

The potential presence of metabolic syndrome was evaluated for patients with laboratory values. According to IDF criteria, if:

- in addition to central obesity (defined as waist circumference of ≥ 94 cm for Europid males and ≥ 80 cm for Europid females) any two of the following four factors could be confirmed, namely:
- triglyceride concentration: > 1.7 mmol/L, or pharmacological therapy due to elevated triglycerides;
- low high-density lipoprotein (HDL) cholesterol concentration: < 1.03 mmol/L for males and < 1.29 mmol/L for females, or pharmacological therapy due to low HDL cholesterol;
- elevated blood pressure: systolic value of ≥ 130 mmHg or diastolic value of ≥ 85 mmHg, or prior diagnosis of hypertension, or pharmacological therapy due to elevated blood pressure;
- elevated fasting blood glucose concentration: ≥ 5,6 mmol/L, or prior diagnosis of T2DM;

metabolic syndrome can be diagnosed (Alberti *et al*., 2006; Zimmet *et al*., 2007).

### Statistical analysis

Results of the research study were processed with descriptive and mathematical statistical methods using IBM SPSS 22.0 (Statistical Package For Social Science) and Microsoft Excel software. Descriptive statistics included the calculation of mean, standard deviation (SD) and confidence intervals (CI) values. In the absence of a normal distribution of variables, non-parametric tests, Spearman’s rank correlation coefficient and Wilcoxon’s test were used to assess the relationships between groups, while the quality variables and the relationship between metabolic syndrome and carbohydrate metabolism disorders were evaluated with the binominal logistic regression and the Chi-squared test were calculated. The level of significance was set at 5% (*P* < 0.05).

## Results

### Demographic data and medical history

A total of 854 adults fulfilled the inclusion criteria and the FINDRISC questionnaire was filled out for 64.52% of them. Totally, 200 males (36.30%) and 351 females (63.70%) participated in the study. The average age was 42.39 years ± 16.104 SD (95% CI: 41.04–43.74) and the average score on the FINDRISC questionnaire was 8.56 points ± 4.64 SD (95% CI: 8.17–8.95). And 26.70% of the patients (*n* = 147) in our sample were found to be at moderate or high risk. In our sample, 56.08% were regular smokers, 35.75% were receiving treatment due to hypertension and only 7.26% reported less than 30 minutes physical activity (work) per day, and 50.64% reported a positive family history for diabetes. (Table 3)

**Table 3.** Presentation of results in the whole sample and risk groups

Examined factors	Results by all patients *n* = 551	Results of moderate- and high-risk patients *n* = 74	*P*-value
**Age**	42.39 years ± SD 16.104 (95% CI 41.04–43.74)	56.64 years ± SD 11.606 (95% CI 53.95–59.32)	0.016
**Sex**	Male: 36.30% (*n* = 200) Female: 63.70% (*n* = 351)	Male: 29.7% (*n* = 22) Female: 70.3% (*n* = 52)	0.049
**FINDRISC score**	8.56 points ± 4.64 SD (95% CI 8.17–8.95)	14.51 points ± SD 2.23 (95% CI 14.0–15.03)	0.092
**Smoking**	Smoker: 56.08% (*n* = 309) Non-smoker: 43.92% (*n* = 242)	Smoker: 36.5% (*n* = 27) Non-smoker: 63.5% (*n* = 47)	0.266
**Hypertension**	Yes: 35.75% (*n* = 197) No: 64.25 (*n* = 354)	Yes: 83.8% (*n* = 62) No: 16.2% (*n* = 12)	0.690
**Family history of diabetes mellitus**	Negative: 50.64% (*n* = 279) Positive: 49.36% (*n* = 272)	Negative: 41.9% (*n* = 31) Positive: 58.1% (*n* = 43)	0.230
**Physical activity** **(min. 30 min/day)**	Yes: 92.74% (*n* = 511) No: 7.26% (*n* = 40)	Yes: 85.1% (*n* = 63) No: 14.9% (*n* = 11)	0.456
**BMI (kg/m** ^**2**^ **)**	27.01 kg/m^2^ ± SD 5.92 (95% CI 26.51–27.507)	33.09 kg/m^2^ ± SD 5.51 (95% CI 31.81–34.37)	0.991
**Waist circumference (cm)**	94.11 cm ± SD 16.80 (95% CI 92.71–95.52)	110.35 cm ± SD 16.34 (95% CI 106.56–114.14)	0.524
**Metabolic syndrome**		Negative: 29.73% (*n* = 22) Positive: 70.27% (*n* = 52)	0.038

SD = standard deviation; CI = confidence interval; BMI = body mass index

### Results of the diabetes risk analysis

Based on the FINDRISC scores, 76.5% of male patients (*n* = 153) were classified as low or slightly elevated risk, while 23.5% (*n* = 47) were classified as moderate or high risk. Results of the female patients of the sample were as follows: 71.51% (*n* = 153) were classified as low or slightly elevated risk, while 28.49% (*n* = 100) were considered to be at moderate or high risk. A significant (*P* < 0.001) increase in diabetes risk was confirmed for patients above 45 years of age.

In sample, the average BMI was 27.01 ± 5.92 SD kg/m^2^ (95% CI: 26.515–27.507). Only 5.08% were moderately or mildly thinness. And 35.54% had BMI values in the normal range, while 30.1% were considered to be overweight. And 29.3% were diagnosed with obesity of varying degrees. While in the low-risk group only 26.31% of patients (*n* = 60) were overweight or obese, this ratio rose to 86.52% (*n* = 77) in the moderate-risk group. The high-risk group consisted entirely of overweight and obese patients (*n* = 58). Significant correlation (r_rho_: 0.627; *P* < 0.001) was with Spearman’s test confirmed between body mass and the increase in diabetes risk.

### Incidence of carbohydrate metabolism disorders

According to the results of the FINDRISC questionnaire, 147 patients in our sample were found to be at moderate or high risk. During the study period, three patients died, two patients refused the participation in the study, seven patients moved from the town, and one patient was not included due to active oncological treatment. Thus, a total of 134 patients were eligible for screening. Among them, 59 patients have never presented for laboratory examination in spite of having been offered several times to do so, although none of them has explicitly refused the offered screening examination. The mean age was 56.13 years ± SD 12.319 years (95% CI 53.30–58.97). High- and moderate-risk individuals received oral and written information and referred for laboratory tests which were completed by 55.22% (74 patients).

#### Incidence with HbA_1c_

Based on the ADA criteria, normal carbohydrate metabolism status was revealed in 43.24% of patients (*n* = 32); this rate was 71.62% (*n* = 53) when the WHO criteria were used. Pre-diabetes was proved in 44.60% according to the ADA criteria, while this rate was only 16.22% according to the WHO criteria. The incidence of diabetes was the same, being 12.16% in both cases, which was due to the identical value range.

#### Incidence with OGTT

**Figure 1.**
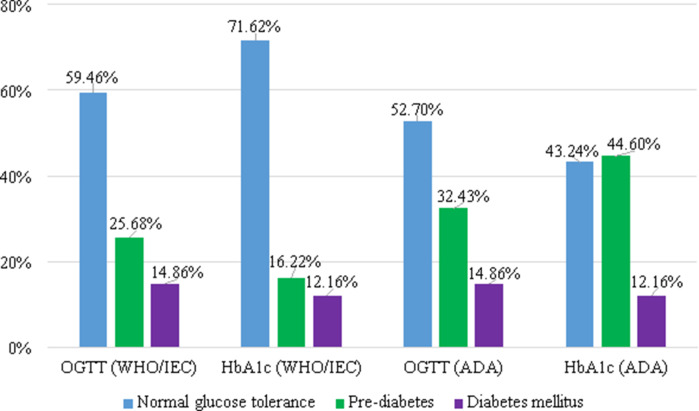
Incidence of carbohydrate metabolism disorders: comparison of different laboratory methods and diagnostic criteria. WHO = World Health Organization; IEC = International Expert Committee; ADA = American Diabetes Association; OGTT = oral glucose tolerance test; HbA_1c_ = glycated haemoglobin.

No significant difference was found among patients with normal carbohydrate metabolism status (59.46% versus 52.70%). And 25.68% and 32.43% of patients was proven to have pre-diabetes according to the WHO and ADA criteria, respectively. Both criteria systems revealed diabetes in 14.86% of patients, in whom diabetes had not been diagnosed previously (Figure 1). And 13.51% of patients had isolated IGT, which definitely supports the indication for OGTT among patients who are in the risk group.

The difference between the measured results of the two screening methods was proven with Wilcoxon’s test. The most significant difference between the results measured by the two criteria systems was detected in the HbA_1c_ values (Z: −3.838 *P* < 0.001). (Table 4).

**Table 4.** Differences of measurement results between ADA and WHO/IEC criteria

	OGTT (ADA) versus OGTT (WHO/IEC)	HbA_1c_ (WHO/IEC) versus HbA_1c_ (ADA)	HbA_1c_ (WHO/IEC) versus OGTT (WHO/IEC)	HbA_1c_ (ADA) versus OGTT (ADA)	HbA_1c_ (ADA) versus OGTT (WHO/IEC)
Z	−1.890	−3.838	−1.961	−0.600	−3.285
*P*-value	0.059	0.000	0.050	0.549	0.001

OGTT = oral glucose tolerance test; HbA_1c_ = glycated haemoglobin; ADA = American Diabetes Association; WHO = World Health Organization; IEC = International Expert Committee

Based on the practice and guidelines applied in Hungary, the WHO criteria system was used for further investigation of positive cases. Patients with positive HbA_1c_ both for pre-diabetes and diabetes mellitus were taken into account. We investigated which known diabetes risk factors affected the carbohydrate metabolism disorders. The strongest predictor of carbohydrate metabolism disorders were age (*P* = 0.039) and sex (*P* = 0.049). The weakest predictors were hypertension (*P* = 0.690) and BMI (*P* = 0.991) (Table 3).

### The relationship between metabolic syndrome and HbA_1c_

Based on the IDF criteria, 70.27% of patients (*n* = 52) with laboratory results were confirmed to have metabolic syndrome. The rate was higher (65.39%) among our female patients. Further analysis of the data of patients with normal carbohydrate metabolism or pre-diabetes concentrated on the question whether the presence of metabolic syndrome can induce an increase in HbA_1c_. The presence of metabolic syndrome induced a 0.2% increase in HbA_1c_ values in normal carbohydrate metabolism groups (*P* = 0.038) which was not affected by age. At the same time, we did not confirm same differences in carbohydrate metabolism disorders group.

## Discussion

### Main findings

Our research highlights a highly significant public health issue. In addition to carbohydrate metabolism disorders, we successfully identified several risk factors affecting our patients. We had a chance to research the very high incidence of diabetes and its precursor states in a disadvantaged population and hope to use our experiences to tailor further preventative efforts and methods. In fact, at least 30% of the patients involved in our study (or even higher ratio, depending on the laboratory diagnostic criteria) was proven to have some kind of carbohydrate metabolism disorder. The research study provided unequivocal proof that low levels of education and poor socio-economic conditions greatly affect morbidity, mortality and the effectiveness of preventative efforts in primary care. Locally available screening tests could significantly increase participations rates and free economically disadvantaged patients from incurring undue expenses.

### Interpretation of the study results

When comparing the prevalence of diabetes and its precursor states to results from earlier studies conducted in Hungary, we detected a higher prevalence of diabetes. In 2010 and 2011, Winkler et al. used the same two-stage method and GPs working in primary care to screen for diabetes. And 22.846 patients participated in glucose tolerance test. And 46.53% of patients were diagnosed with manifested diabetes mellitus or one of its precursor states (IFG: 14.1%, IGT: 24.8%, manifested diabetes: 7.6%) (Jermendy *et al*., 2010a; Winkler *et al*., 2013). Our results showed higher rates of newly diagnosed diabetes mellitus with both laboratory test methods and criterias. However, in our study, the rate of pre-diabetes diagnosed was near equivalent among patients with a positive laboratory result. Another negative fact is that the mean age of the patients participating in our study was lower than that of the participants of a study conducted in Hungary 10 years earlier (when it was 50.4 years), and our results were proven to be worse now. It is may be due to the worse socio-economic status of the habitants of the town. Results align well with previous data from literature showing a significant increase in diabetes risk among people over 45 years of age, underlining the importance of initiating regular screening in this age group. Since the incidence of isolated IGT was significantly elevated, at-risk patients must be referred for OGTT even if they have negative FPG results. The HbA_1c_ test is both useful and informative; therefore, it is highly recommended to add it to the battery of screening tests – in addition to FPG or OGTT – prescribed for high-risk patients, while keeping in mind the diagnostic limitations of this method (Sequeira and Poppitt, 2017). In addition, we also analysed whether the presence of metabolic syndrome can induce an increase in HbA_1c_ values. Our results showed that, in normal carbohydrate metabolism status, HbA_1c_ values were on average 0.2% higher in patients with metabolic syndrome, which did not come from age. This finding may prove useful in everyday practice because it can help determine the patient’s actual carbohydrate metabolism status in borderline cases where the two laboratory diagnostic tests do not provide an unambiguous definition of the patient’s carbohydrate metabolism status. Therefore, it is recommended to test for the presence of metabolic syndrome and then evaluate HbA_1c_ values based on the test results. With regard to the use of FPG (based on WHO criteria) and HbA_1c_ (based on ADA criteria) test, our results of a 3.5 times difference in incidence were almost identical to results published in the international scientific literature (Rosella *et al*., 2015; Kim *et al.*, 2016; Unwin *et al*., 2017). It is important to highlight that, in addition to carbohydrate metabolism disorder, participants in our research study had at least one additional cardiovascular risk factor which further increases the risk of premature death and disability. A significant proportion (56.08%) of participants were smokers. According to WHO statistics for the year 2017, 26% of the population of Hungary were smokers; thus, the rate of smoking in our study population was significantly higher than the national average (World Health Organization, 2019). Our results on overweight and obesity rates were in line with data recorded in 2014 in the course of the Hungarian Diet and Nutritional Status Survey (OTÁP) (Rurik *et al*., 2016; Erdei *et al*., 2017).

### Implications for clinical practice

The first step of screening tests should be the identification and assessment of risks which should be included in the regular health status assessment (performed every 2 or 5 years according to Hungarian regulations) (Jermendy *et al*., 2010b). HbA_1c_ as a screening test is both useful and informative and should be added to the OGTT – especially in the case of high-risk patients – and incorporate into the assessments used in everyday general practice (Twohig *et al*., 2018). Health statistics from the region – including the affected municipality – show higher mortality due to cancer and cardiovascular diseases. It is likely that diabetes significantly contributes to the elevated rates; therefore, prevention and early diagnosis are important. If carbohydrate metabolism disorders are diagnosed in the precursor states, with the appropriate cooperation of the patient, it may be possible to reverse or at least delay the development of diabetes mellitus and its complications. For patients with moderate- or high-risk for diabetes mellitus, following up screening with appropriate interventions is of primary importance. Based on the National Institute for Health and Care Excellence (NICE) guidelines revised in 2017, part of the prevention of progression is providing assistance to the affected patients via programmes promoting lifestyle changes. In addition to conventional lifestyle change items already used in general practice, these programmes also include the mapping of typical diets in the local region, examination of options for weight loss and physical activity and providing the patient with all this supplementary information, including the locations and contact information of facilities and the competencies of participating professionals, etc. If the availability of intensive lifestyle change programmes is limited, then priority access should be offered to patients with the highest risk (FPG: 6.5–6.9 mmol/L or HbA_1c_: 6.2–6.4%). If the assistance does not result in satisfactory change and the patient shows progression, or the patient is unable to participate in the programmes and their BMI exceeds 35 kg/m^2^, then institution of metformin therapy should be considered (National Institute for Health and Care Excellence, 2017).

### Strengths and limitations

The greatest limitation of the research was the poor health culture and health awareness of our patient population. It was hard to motivate our patients to participate in screening tests and to counteract the negative perceptions accompanying the potential diagnosis of disorders. Although the participation rate of our patients was higher than that of the general population of Hungary, it was still below our expectations. Many patients found it difficult to cover the travel expenses the laboratory tests required, but the office of the general practice was not a suitable location for blood draws.

### Conclusion

Due to the public health significance of diabetes mellitus as well as its health economic consequences, professionals working at a general practice should be up to date regarding the screening methods. Intervention performed at the right time using the right method is a basic expectation for primary healthcare. When comparing the screening methods, significant differences were revealed between the WHO and the ADA criteria systems regarding prevalence. Both methods and criteria systems have advantages and disadvantages. As the population of Hungary is considered to have a high risk for cardiovascular diseases, and considering the fact that the majority of the patients involved in our study had at least one cardiovascular risk factor in addition to carbohydrate metabolism disorder, the application of HbA_1c_ during screening would be very important. Recommendations in the literature also suggest that evaluation of the results should be performed according to the ADA guidelines, as we should applied that these patients have higher cardiovascular risks; thus, stricter target values are required. Once carbohydrate metabolism disorder has been diagnosed, choosing the appropriate intervention, providing access to lifestyle change assistance programmes and if necessary, institution of medicinal therapy are all tasks of primary importance for primary care professionals aiming to prevent further progression. However, efficient implementation requires sufficient human resources, appropriate infrastructure as well as adequate time for communicating with/treating the patients, which are inadequate quantity currently in primary care in Hungary.
